# Abortion in Domesticated Animals

**Published:** 1897-02

**Authors:** Charles F. Dawson

**Affiliations:** Veterinary Inspector, Bureau of Animal Industry


### ABORTION IN DOMESTICATED ANIMALS.

By Charles F. Dawson,
VETERINARY INSPECTOR, BUREAU OF ANIMAL INDUSTRY.

There are perhaps few veterinarians or stock-raisers who have not at some time encountered instances of this trouble in the domesticated animals. Authors on veterinary matters generally recognize two kinds of abortion.[1] The first, and probably the least in importance so far as this paper is concerned, is known professionally as sporadic abortion. We believe that a large number of the cases coming under this head are, as it is claimed for them, due to accidental causes or external influences. Some of them are very likely due to the ingestion of certain substances which have a specific influence upon the genital organs, acting directly upon them through the parturition centre. Other ingesta may, by causing violent purgation from irritation of the intestinal walls, have their influence upon the uterine muscular tissue by induced uterine hyperæmia.

In support of the views held by many practitioners that cattle, horses, and sheep do at times eat along with their fodder certain plants which have abortifacient properties, I quote from a note by the late Professor Joseph Leidy,[2] in which he says: "Some years ago a physician living in the lower part of our city submitted to me for determination a root-fragment which he said was used in the circle of his practice in a rural district as a domestic emmenagogue. Not recognizing the root, he afterward brought to me a portion of the stem and leaves of a plant which proved to belong to the common ironweed (*Vernonia noveboracensis*), growing abundantly in the meadows. It has occurred to me that if this plant has the qual-

[1] In this paper, and according to the general custom among veterinarians, the term abortion is used as indicating a premature expulsion of the fœtus regardless of the question of its viability.

[2] Journal of Comparative Medicine and Surgery, vol. viii. 1887.

ity attributed to it, it may, when cut among hay and fed to cattle, be one of the causes of abortion among cows."

Because it is well known that ergot, by reason of its action upon the vasomotor nervous system, can cause the uterus to contract upon its contents with sufficient force to expel them, and because of the frequent occurrence of this fungus in the fodder of animals, it has been reported as being a factor in causing a large number of cases of sporadic abortion. There are, however, records of numerous experiments where ergot has been fed in large quantities to pregnant cattle without causing them to abort. If we accept that ergot has the same systemic effects in all animals we should conclude that either the doses given were not sufficiently large and frequent or that the ergot used did not contain the ecbolic principle. We know that ergot does exert its physiological action upon cattle, because of a notable outbreak of ergotism which occurred several years ago in Kansas.[1] Great excitement prevailed among stockmen at the time, they fearing that they had to do with an outbreak of foot-and-mouth disease. An investigation by the department revealed the fact that the hay upon which the animals had been feeding contained large quantities of ergotized rye. The characteristic effects of the drug were present; the extremities became gangrenous and sloughed away.

The literature of the subject of abortion teems with contradictory statements both from members of the veterinary profession and from laymen. The agricultural societies of Great Britain have appointed numerous committees to investigate the disease, with the result that it seems to-day that very little more is known about it than formerly.

The alleged causes of sporadic abortion are very numerous, indeed, and it would seem that the number of conditions which might cause an animal to abort is limited entirely by environment and existing circumstances. We can very well understand that if a pregnant animal were jammed in a stall or doorway or were to receive a violent blow upon the abdomen, or were subject to external violence of any kind, that the placental attachments could thereby be ruptured. This happening the fœtus would die, become a foreign body, and be expelled.

Many arguments, pro and con, have been made regarding another cause of sporadic abortion—that of sympathy. We can conceive that an animal may, through the organs of special sense, receive a

[1] First Annual Report, Bureau of Animal Industry, 1884.

mental shock sufficiently severe to be reflected to parts of the sympathetic nervous system. While we have no data to prove that sympathy ever causes an animal to abort, and while we do not believe that the sympathetic nervous system is as highly developed in the lower animals as it is in man, we have the familiar old sight that a cow roughly treated will not "give down" her milk, and that the presence of the calf will cause the milk to flow.

It is an unquestioned fact that in human practice the uterus of the puerperal patient may be made to contract by the simple application of the newborn to the mother's breast. In fact, this knowledge is made use of in some instances where the uterus seems slow to contract. Can we have any better evidence of the close bond of sympathy existing between the various parts of the nervous system? If the slight stimulus exerted by the application of the young to the mammæ, or the mere presence of the young, so affects the nervous organization of the mother as to cause a "flow" of milk, or a contraction of the uterus, why might we not conclude that in severe nervous shocks, such as sudden fright, being chased by dogs, the sight and odor of blood, etc., we have stimuli sufficiently severe to cause the uterus to contract with sufficient force to expel its contents?

We must remember that in all the domesticated animals the sense of smell is much more highly developed than in ourselves, and that because an odor does not to us seem to be of sufficient intensity to warrant us in believing that it could exert any great influence upon the sympathetic nervous system, is no reason at all for believing that the same argument would be true in the case of the lower animals.

As before stated, we say that all cases of abortion which result from kicks, blows, disgusting sights, odors, etc., are due to external causes. Now we may have sporadic abortion occur from what we are pleased to term internal or systemic causes. Under this head we would put those cases which could occur from the ingestion of food containing plants which have ecbolic properties. We may also have as a cause of sporadic abortion certain conditions of the animal's system. Any severe disease may so affect the pregnant animal as to cause it to abort. A severe lung-disease in which the respiratory area is greatly lessened, causing an imperfect oxygenation of the blood, would make itself felt at the point of greatest tissue-change, the uterus in this case, and would be very apt to start up a revolution in that organ.

A badly ventilated stable containing many animals could, by virtue of the gradual accumulation of the carbon dioxide and other poisonous exhalations from the animal body, be the cause of abortion. This, however, we believe to be a very infrequent cause, as most stables have sufficient ventilation to prevent the accumulation of such gases in poisonous qualities.

Cases of abortion could occur from a displacement of the uterus, and by any cause which would operate to prevent the natural enlargement of that organ, such as fibrous adhesions to other structures.

On account of the immense volume of the stomachs in the cow, and of the cæcum and colon in the mare, it is easily understood how, in cases of acute indigestion, when those viscera are greatly distended with gas from the fermentation of their food-contents, they can by encroaching upon the gravid uterus exert an influence upon that organ and cause it to expel its contents.

Theoretically, any disease or condition which impoverishes the blood also impoverishes the uterine structures, and we probably have some cases of abortion due to this state of the blood, or anæmia. Therefore, Texas cattle-fever, which has been shown by Dr. Theobald Smith to be due to a parasite which lives at the expense of the blood, should cause a pregnant cow to abort, provided she had the disease in a severe form.

Since writing the above regarding the influence of anæmia brought about in the blood of cattle suffering with Texas cattle-fever, I have received the following evidence that the theory is a correct one. Col. R. J. Redding says in a letter addressed to Dr. D. E. Salmon: "Mr. Wing desires me to state a circumstance in regard to the outbreak of Southern cattle-fever in Greene County that he overlooked in writing to you the other day, viz., of the cows that were attacked that were pregnant, all, excepting one, aborted, and of several that were nearly due to calve or a few days overdue the calf in every case was dead."

On the contrary, abortion may be caused by an opposite condition—that of plethora, where we have an abnormal accumulation of food-principles in the blood, and where, from the decreased activity of the secreting and excreting organs, the abnormal accumulations are not removed.

Sudden changes of temperature can cause abortion by a central determination of the peripheral blood. In order to understand this condition, better known as a "congestive chill," we must recognize that as soon as an animal becomes pregnant the uterus becomes the seat of a most extraordinary tissue-metamorphosis; that in order for these great changes to take place the uterine blood-supply must be greatly increased. Those who have studied the uterine bloodvessels of a non-pregnant animal will remember that the uterine arteries are not relatively large. In the pregnant state, however, the reverse will be found to be true. The former small, tortuous arteries are now much enlarged, carrying very much more blood than they did formerly. So when from a sudden blanching of the skin and superficial structures the blood is driven to the internal organs the uterus gets a large share, and becomes congested, the delicate lining of the walls of the arteries in the placental structures becomes injured, and the maternal connection of the fœtus is severed.

(To be continued.)

